# ^18^F‐sodium fluoride positron emission tomography may help determine better treatment for thigh pain after hip arthroplasty—A case report

**DOI:** 10.1002/ccr3.2920

**Published:** 2020-05-26

**Authors:** Shinya Nakamura, Shigehito Uezono, Yuko Nagai, Masakazu Kanetaka, Kei Wagatsuma, Kenji Ishii, Takeshi Kumakawa, Kensuke Yasue, Yorito Anamizu, Fumiaki Tokimura, Tsuyoshi Miyazaki

**Affiliations:** ^1^ Department of Orthopaedic Surgery Tokyo Metropolitan Geriatric Hospital and Institute of Gerontology Tokyo Japan; ^2^ Team for Neuroimaging Research Tokyo Metropolitan Geriatric Hospital and Institute of Gerontology Tokyo Japan

**Keywords:** atypical femoral fracture, hip arthroplasty, loosening, NaF, Positron emission tomography

## Abstract

Thigh pain after hip arthroplasty is multifactorial; uncovering its etiology is paramount for optimal treatment. This is the first case where ^18^F‐sodium fluoride positron emission tomography substantially helped in diagnosing the post‐hip arthroplasty persistent thigh pain and appropriate treatment selection. This imaging modality warrants further study and more widespread application.

## Graphical abstract

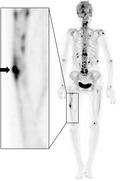

## 1 INTRODUCTION

Hip arthroplasty is a surgical procedure in which the hip joint is replaced by a prosthetic implant. Hip arthroplasty can be performed as a total replacement or a hemi‐replacement and is currently one of the most common orthopedic operations with long‐term patient satisfaction. Such orthopedic joint replacement is generally conducted to provide relief from arthritic pain or to treat displaced femoral neck fractures in the elderly. Total hip arthroplasty consists of replacing both the acetabulum and the femoral head, whereas hemiarthroplasty generally only replaces the femoral head.

Thigh pain is a significant complication following successful hip arthroplasty. The incidence of thigh pain reported in the literature ranges from 1.9% to 40.4%.^1^ In most cases, reported symptoms are mild to moderate, resolve spontaneously, or do not progress, and require little to no therapeutic intervention. However, persistent thigh pain may be a source of dissatisfaction or may present as severe, disabling pain. Understanding potential etiologies, clinical presentation, and critical elements of the diagnostic evaluation is paramount for selecting the optimal treatment modality. Factors that have been associated with the occurrence of thigh pain following hip arthroplasty include bone type, larger stem size, uncemented femoral components, straight femoral stem design, stem material composition, femoral component instability, and loosening.^2^, ^3^, ^4^ Other causes including infection, stress fracture, and spinal problems, need to be ruled out.

Positron emission tomography (PET), which is noninvasive and usually painless, uses a positron emitter and a scintillator to detect annihilation photons. By providing molecular information, PET may identify changes at the cellular level and detect the early onset of disease before it is evident on other imaging modalities. The recently developed ^18^F‐sodium fluoride PET/computed tomography ([^18^F] NaF PET/CT) is a more accurate diagnostic tool for specific bone disorders. Most of the NaF transported to the bone is only retained following a single pass of blood, making it a suitable radiopharmaceutical agent for assessing subtle changes in bone metabolic activity.^5^, ^6^

Here, to the best of our knowledge, we present the first case where NaF PET/CT substantially assisted the diagnosis of persistent activity‐limiting thigh pain following hip arthroplasty, leading to the selection of the most appropriate treatment strategy.

## 2 CASE PRESENTATION

**Figure 1:**
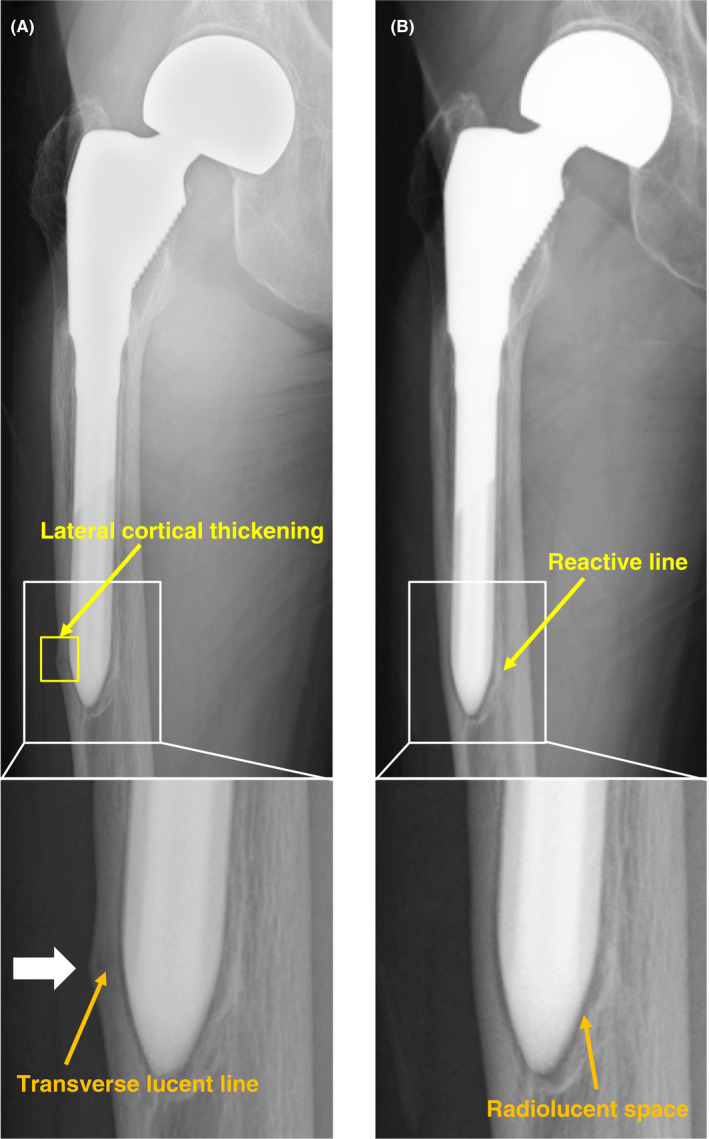
Varus femoral component with beaking and an obscure transverse lucent line a little above the stem tip. A, Frontal femur radiography obtained when the patient first visited to our hospital complaining of right severe thigh pain showed a lucent area around the stem with subtle thickening and beaking of the lateral cortex near the distal stem tip (white arrow). A higher magnification image of the framed area is shown in the lower panel. Yellow and orange arrows indicate lateral cortical thickening and an obscure transverse lucent line, respectively. B, Radiograph obtained 3 y earlier showed no periosteal thickening of the lateral cortex a little above the stem tip. A higher magnification image of the framed area is shown in the lower panel. Yellow and orange arrows indicate a reactive line and a radiolucent space, respectively

**Figure 2:**
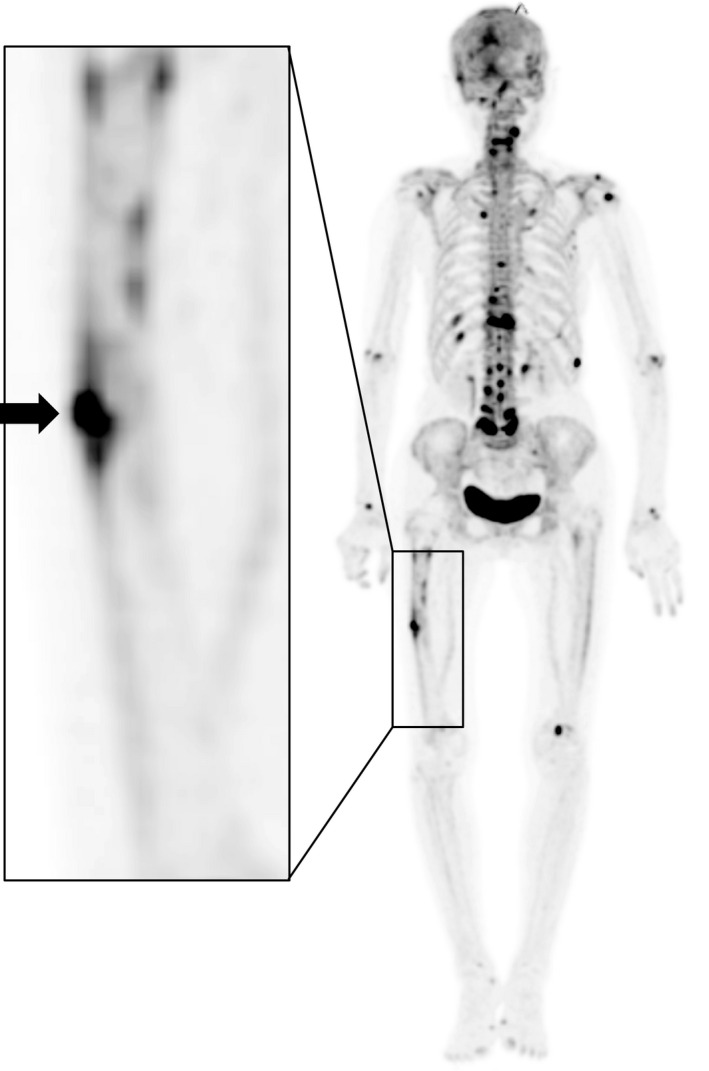
^18^F‐sodium fluoride (NaF) positron emission tomography image showing high [^18^F] NaF uptake only at the beaking of the lateral cortex a little above the stem tip (black arrow) in contrast to low uptake surrounding the femoral component. A higher magnification image of the framed area is shown in the left panel

**Figure 3:**
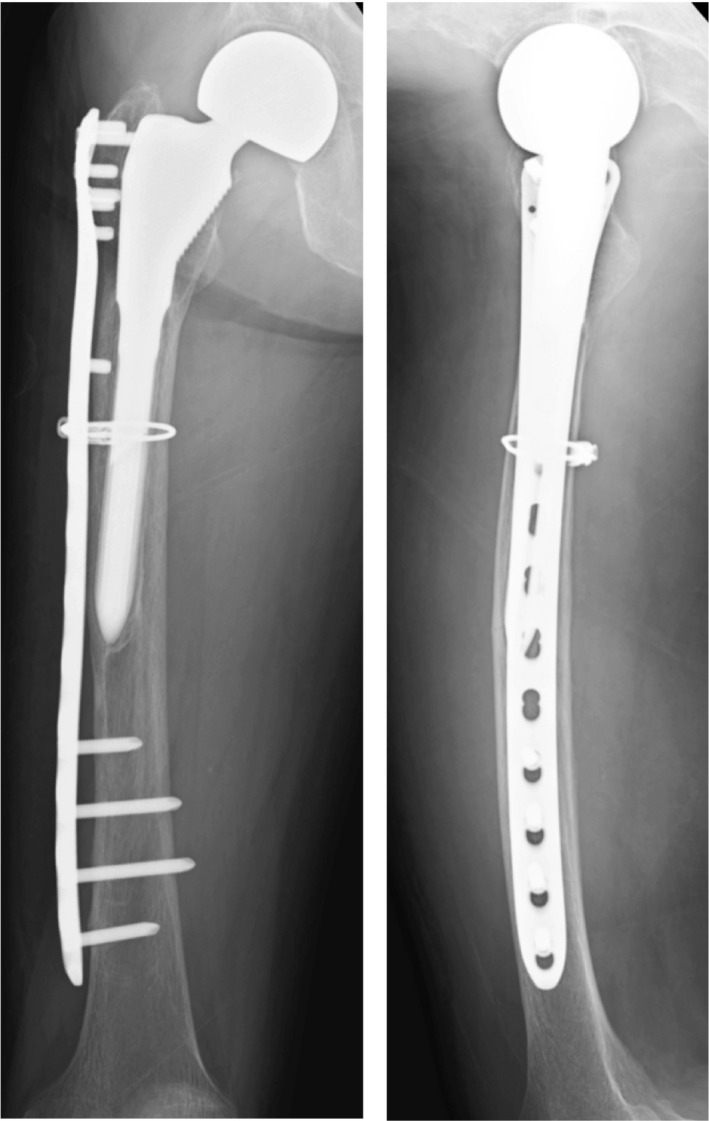
Radiographs of the right femur (anteroposterior and lateral) obtained 1 mo following preventive open reduction and internal fixation were performed

**Figure 4:**
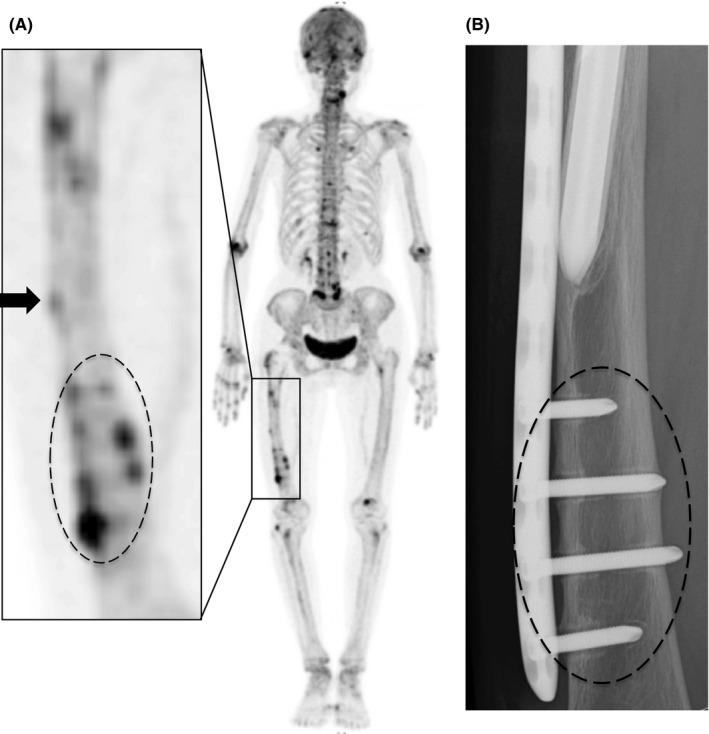
A, ^18^F‐sodium fluoride positron emission tomography (NaF PET) image obtained 1.5 y following surgery showed that the high NaF uptake at the beaking of the lateral cortex a little above the stem tip was dramatically reduced (black arrow). There was high‐intensity area around the distal screws below the stem end (dotted circle) A higher magnification image of the framed area is shown in the left panel. B, Radiograph of the right femur (anteroposterior) taken at the same time with the NaF PET/CT scan showed radiolucent zones in the corresponding area of the NaF PET image. (dotted circle)

A 77‐year‐old woman visited our hospital with no history of trauma and complained of right thigh pain. Although her pain initially occurred late during activity and was resolved with rest, it finally became more constant until it was present at rest (Table 1). She received right cementless bipolar hip hemiarthroplasty (BHA) in her local hospital 18 years earlier due to a right femoral neck fracture and had a history of 2‐year alendronate (35 mg/wk) use. Radiography showed a radiolucent space surrounding the distal portion of the stem with reactive lines, accompanying localized lateral cortical thickening and an obscure transverse lucent line just above the stem tip (Figure 1A). At 3 years prior to this episode, she visited our hospital for left‐sided sciatic pain with magnetic resonance imaging only indicating mild spinal canal stenosis of the L5‐S1. She presented no pain or other symptoms in her right leg during that time. Therefore, we accessed the conventional anteroposterior radiographs of the hip joint taken at the time of the lumbar magnetic resonance imaging scan. Reactive lines and radiolucencies were already apparent, and there were no signs of progressive component migration during these past 3 years (Figure 1A, B), even though no beaking of the lateral cortex was observed in this earlier radiograph (Figure 1B). One possible cause of her severe thigh pain could be aseptic loosening of the stem as plain radiography showed a periprosthetic zone of radiolucency around the bone‐implant interface. Another possibility was that the incomplete stress fracture with specific features reflective of atypical femoral fractures (AFFs) was responsible for the painful hip arthroplasty. Tam et al^7^ reported that comparing the pattern of uptake with previous bone scintigraphy studies is important to determine loosening. A higher intensity around the prosthesis compared with the previous studies may raise the suspicion of aseptic loosening. Conversely, should no increased uptake on delayed bone scintigraphy exist, significant loosening is unlikely. Furthermore, acute fractures or unstable and unfused old fractures were reported to show increased uptake on bone scintigraphy. Furthermore, we examined her right thigh with [^18^F] NaF PET/CT to investigate the cause of the pain. NaF PET/CT demonstrated focal intense tracer uptake at the beaking of the lateral cortex; however, only mild radiotracer uptake was observed even in the radiolucent space around the distal portion of the stem (Figure 2), suggesting that the cause of the pain was probably due to AFF‐like incomplete stress fracture. Hence, we performed open reduction and internal fixation (ORIF) using a locking plate. We chose a reversed locking compression plate for the distal femur (LCP distal femur plate; Depuy Synthes) for the contralateral side and an LCP cable system (Figure 3). Alendronate treatment was discontinued and teriparatide (20 µg/d) was started following surgery. We prescribed a rehabilitation program including immediate, gradually augmented weight‐bearing, and she reported alleviation of pain a couple of weeks following surgery. Unfortunately, bone healing such as callus formation or fracture line disappearance could not be confirmed on radiograph due to overlapping incomplete fracture site and LCP plate. One and half year following surgery, we reperformed NaF PET/CT scan again, and it showed a markedly reduced NaF uptake at the lateral cortex near the distal stem tip (Figure 4A). Unexpectedly, the high intensity around the distal screws was observed in contrast to low uptake along the distal stems. In addition, anteroposterior radiography of the right femur performed simultaneously, also showed radiolucent zones around the distal screws (Figure 4B). These findings indicated that hardware micromotion may have induced high bone metabolic activity, resulting in high concentrations of NaF on PET around the distal screws. She is currently (2.5 years postoperatively) under observation and ambulates independently with no complications (Table 1).

**Table 1:** Timeline of pain scale and walking aid

	T0	T1	T2	T3	T4	T5	T6
Pain scale	0/10 during activity 0/10 at rest	7/10 during activity 1/10 at rest	8/10 during activity 3/10 at rest	3/10 during activity 1/10 at rest	2/10 during activity 0/10 at rest	0/10 during activity 0/10 at rest	0/10 during activity 0/10 at rest
Walking aid	No aid used	Walking stick	Walking stick	Walking frame	Walking stick	No aid used	No aid used
Aggravating factors		Prolonged standing, and climbing stairs	Prolonged standing, climbing stairs, active abduction of the hip, and side‐lying positions	Prolonged standing, and climbing stairs	Prolonged walking		
Relieving factors		Medication (NSAIDs) and still sitting	Minimal pain relief with medication (NSAIDs)				

Notes

Pain at rest and during activity was assessed by the visual analog scale (VAS).

T0, three years prior to the initial presentation (when the patient visited the hospital for the left‐sided sciatic pain); T1, the initial presentation; T2, at the time of the first NaF PET scan (immediately before operation); T3, immediately following operation; T4, during the time of rehabilitation; T5, at the time of the second NaF PET scan (1.5 y following operation), T6, 2.5 y following operation.

## 3 DISCUSSION

We described a case of persistent severe thigh pain that occurred in a patient who had previously undergone BHA for femoral neck fracture treatment. As diagnosing the cause of the severe thigh pain proved challenging, we used NaF PET/CT to determine the treatment strategy. To our knowledge, this is the first case where NaF PET/CT substantially helped us in concluding that the severe thigh pain was due to an incomplete stress fracture just above the stem tip, and not due to aseptic loosening. Treatment with rigid internal fixation for AFF‐like incomplete fracture and improvement of the bone metabolism using teriparatide successfully led to pain alleviation and walking ability restoration.

The etiology of thigh pain is often multifactorial and can be generally categorized into factors related to micromotion at the bone‐prosthesis interface, excessive stress transfer to the femur; host bone morphology; endosteal/periosteal irritation; unnoticed intraoperative fracture; and a mismatch in Young's modulus of elasticity that increases the structural rigidity of the prosthetic stem relative to the femur. These factors are likely interrelated and may stimulate the final common pain mediators, that is, the endosteum and periosteum. Thorough diagnostic evaluation of thigh pain is essential to rule out prosthetic infection, loosening of the stem, stress fracture, or spinal pathology as the primary source. In this report, we found that NaF PET/CT was useful for determining the treatment strategy in thigh pain initially appearing on radiographs as stem loosening. Radiographs showed varus alignment of the femoral stem with associated thinning of the lateral cortex at the distal stem tip (Figure 1A). In addition, radiolucent zones at the bone‐metal interface at the distal part of the stem were observed, leading us to suspect that the severe thigh pain was due to the stem loosening. However, there was no increase in tracer uptake surrounding the femoral component on NaF PET/CT except for the area of beaking (Figure 2). Furthermore, serial radiographs (Figure 1A, B) showed that the smooth 1mm or shorter radiolucent lines at the bone‐implant interface, which were accompanied by a sclerotic line parallel to the lucent area, had not progressed over the past 3 years. These findings suggested that the femoral component was fixed by a fibrous tissue ingrowth. Conversely, beaking of the lateral cortex, which developed and gradually became apparent with corresponding increased tracer uptake on NaF PET, allowed us to diagnose AFF‐like incomplete stress fracture. Under these circumstances, we support the view that no revision surgery should be planned and ORIF would be the best option. Furthermore, the finding that the rigid internal fixation dramatically ameliorated her thigh pain and walking ability in combination with the fact that the NaF uptake around the beaking area was clearly downregulated, probably by the ORIF‐mediated decrease in the tensile stress of the lateral femur (Figure 4) suggest that our diagnosis was accurate and our treatment course optimal.

The five major features of AFFs based on the American Society for Bone and Mineral Research task force reports of 2010 and 2014^8^, ^9^ are (a) fractures with minimal or no trauma, (b) a fracture line originates at the lateral cortex and is substantially transverse, (c) complete or incomplete fractures (involving only the lateral cortex), (d) noncomminuted or minimally comminuted fractures, and (e) localized periosteal or endosteal thickening of the lateral cortex at the fracture site. The minor features include a generalized increase in cortical thickness of the femoral diaphysis, prodromal symptoms such as thigh pain, a bilateral incomplete or complete femoral diaphysis fracture, and delayed fracture healing. However, periprosthetic fracture is excluded from this definition,^8^, ^9^ but in some cases, specific features of AFFs have been observed around the periprosthetic region,^10^, ^11^, ^12^ as was also the case here. Although this kind of fracture is not included in the AFF definition, it may have the same or similar pathology. We need to consider it as an AFF, detect it early, and treat it properly even if it occurs in the periprosthetic lesion.

Recently, NaF PET/CT is spreading as a more accurate diagnostic tool for specific bone disorders with the potential to replace conventional bone scintigraphy. Most of the NaF is directly incorporated into the bone following a single pass of blood, making it a suitable agent for the evaluation of subtle bone metabolic changes. By using a quantification parameter such as standardized uptake value (SUV), this method can yield more objective assessments than those obtained with conventional [^99m^Tc] MDP studies. In addition, [^18^F] NaF PET/CT studies are more convenient for patients because the scanning time is less than that with conventional [^99m^Tc] MDP bone scans.

Limitations to the widespread use of PET arise from the high costs of cyclotrons needed to produce the short‐lived radionuclides for PET scanning and the need for specially adapted on‐site chemical synthesis apparatus to produce the radiopharmaceuticals following radioisotope preparation. Therefore, it is important for the patient to be punctual for the appointment and to receive the radioactive material at the scheduled time. Late arrival for an appointment may require rescheduling the procedure for another day. In addition, the results of NaF PET/CT examination are time‐consuming to interpret, containing thousands of images. Also, a great amount of detail is obtained with these examinations, and each area of asymmetric radiotracer uptake needs to be correlated with the CT fusion images. Readers of NaF PET/CT must be aware of the learning curve within image interpretation. These limitations should be overcome in the future.

In this case, we found that NaF PET/CT examination enables us to determine the most appropriate treatment strategy and to provide the patient with accurate information to the patient. Therefore, we should improve our understanding of NaF PET/CT to expand its application and spread its worldwide use. Further investigation involving more patients following arthroplasty will provide new insights into the molecular mechanisms regulating bone metabolism at the bone‐prosthesis interface.

## 4 CONCLUSIONS

Given the high proportion of elderly osteoporotic patients undergoing hip arthroplasty, orthopedic surgeons should consider the possibility of AFFs in patients with thigh pain even when the femoral component is well fixed. Here, it was very difficult to differentiate periprosthetic incomplete stress fracture from aseptic stem loosening as the cause of the severe thigh pain; however, we successfully used NaF PET/CT scans for the precise evaluation of thigh pain that occurred following hip arthroplasty, and the NaF PET/CT images played a decisive role in selecting the optimal treatment course.

## CONFLICT OF INTEREST

None declared.

## AUTHOR CONTRIBUTION

All authors: made substantial contribution to the preparation of this manuscript. SN and SU: wrote the preliminary manuscript. KW and KI: provided PET images. YN, MK, TK, KY, YA, and FT: completed the literature search and found the relevant articles for the discussion. TM: supervised all the work.

## INFORMED CONSENT

Informed consent has been obtained for the publication of the images.
